# Usage and Weekly Attrition in a Smartphone-Based Health Behavior Intervention for Adolescents: Pilot Randomized Controlled Trial

**DOI:** 10.2196/21432

**Published:** 2021-02-17

**Authors:** Erlendur Egilsson, Ragnar Bjarnason, Urdur Njardvik

**Affiliations:** 1 Department of Psychology University of Iceland Reykjavik Iceland; 2 Faculty of Medicine University of Iceland Reykjavik Iceland; 3 Children's Hospital Landspitali University Hospital Reykjavik Iceland

**Keywords:** mHealth, intervention, adolescent, attrition, self-efficacy, mental health, physical activity, young adult, behavior

## Abstract

**Background:**

The majority of adolescents own smartphones, although only 8% of them use health apps. Attrition rates from adolescent mobile health (mHealth) interventions for treating mental health problems such as anxiety and depression are an issue with a high degree of variation. Attrition in mHealth interventions targeting adolescent populations is frequently presented in a two-point fashion, from initiation of the intervention to the end of treatment, lacking more time-specific information on usage and times of attrition. Self-efficacy could provide an avenue to lower attrition rates, although a better understanding of the relationship between mental health factors and time-specific attrition rates is needed.

**Objective:**

The aims of this study were to obtain time-specific attrition rates among adolescents in an mHealth intervention, and to describe the intervention’s usage and feasibility in relation to adolescent self-efficacy levels, and emotional and physical health.

**Methods:**

A single-center randomized controlled public school pilot trial was undertaken with 41 adolescents. Outcome measures were assessed at baseline and after 6 weeks, while in-app activity and attrition rates were continually assessed throughout the intervention period. The primary outcome was attrition based on time and type of in-app health behavior usage, and feasibility of the mHealth app. Secondary outcome measures were self-efficacy levels, depressive and anxiety symptoms, as well as standardized BMI and sleep. Analyses of group mean variances with adjusted α levels through Bonferroni corrections were used to assess main outcome effects.

**Results:**

The attrition from initiation of the intervention to 6-week follow up was 35%. Attrition started in the third week of the intervention and was related to daily time of app usage (*R_t_*=0.43, *P*<.001). The number of average weekly in-app health exercises completed decreased significantly from the first week of the intervention (mean 55.25, SD 10.96) to the next week (mean 13.63, SD 2.94). However, usage increased by 22% between week 2 and the last week of the intervention (mean 16.69, SD 8.37). Usability measures revealed satisfactory scores (mean 78.09, SD 9.82) without gender differences (*P*=.85). Self-reported daily physical activity increased by 19.61% in the intervention group but dropped by 26.21% among controls. Self-efficacy levels increased by 8.23% in the invention arm compared to a 3.03% decrease in the control group.

**Conclusions:**

This pilot study demonstrated the feasibility and usability of an mHealth intervention among adolescent participants. Indications were toward beneficial effects on physical and mental health that warrant further research. Focus on time-specific attrition measures alongside daily times of usage and ways to increase participants’ self-efficacy levels appear to be a promising avenue for research on mHealth interventions for adolescent populations with the aim to ultimately lower attrition rates.

## Introduction

Recent systematic reviews on the global prevalence of psychiatric disorders in children and adolescents have produced varying results ranging from 6.8% to a notably higher pooled rate of 13.4% [1,2]. Emotional disorders as well as significantly distressing subthreshold emotional problems are among the most common psychiatric problems reported in adolescent populations [1-3]. According to the Centers for Disease Control and Prevention, over 6% of US children between 12 and 17 years old have been diagnosed with depression and over 10% have been diagnosed with anxiety disorders [4]. Globally, it is estimated that 10% to 20% of youth experience mental health problems [5-7].

Smartphone ownership is growing fast worldwide. In the United States, smartphone ownership or access among adolescents was estimated at 95% in 2018, representing an increase from 73% in 2015 [8,9]. Similar development is evident elsewhere, with youth smartphone ownership surpassing the 90th percentile in the majority of developed economies [9]. Not only are smartphones widely distributed but people also tend to carry their phones with them, spending an estimated 170 minutes per day using smartphone apps [10]. Some studies indicate that daily smartphone usage among adolescents is often more than 270 minutes [11]. The number of mobile health (mHealth) interventions available has risen steeply in a steady fashion since first appearing roughly a decade ago, with an estimated 325,000 apps available on the market in 2017 [12]. However, only 8% of adolescents use health apps and relatively few studies have documented how they specifically function in adolescent populations [13]. Although mental health problems disproportionally burden minority and lower socioeconomic status groups in terms of receiving evidence-based interventions, smartphones may be used as a tool to diminish such disparities [14,15]. For example, in the United States, adolescent smartphone ownership is not related to gender, race, parental educational levels, or socioeconomic status [9].

Smartphones offer possibilities of a uniquely personalized platform to tailor the many aspects of treatment to individual patients. Patient treatment through support by smartphones or other mobile devices such as tablets, patient monitoring devices, and personal digital assistants have been collectively labeled “mHealth” [16]. mHealth interventions have shown promising cost-effective outcomes related to lowered anxiety and depression symptoms in youth populations despite recurring issues of high attrition rates [15,17-22]. Attrition is here defined as leaving treatment before obtaining a required level of improvement or completion of intervention goals [23,24]. Treatment attrition is common, costly, and important, although varying definitions of the term have challenged research on the matter [17,24].

Studies on mHealth interventions targeting emotional disorders or subthreshold emotional problems in adolescent populations have frequently lacked time-specific data alongside a lack of accurate definitions and analysis of treatment attrition [15,25]. Usability data in mHealth studies targeting adolescent populations are frequently presented with attrition rates from the initiation of intervention, either at the time of recruitment or launch of the intervention’s first section. For instance, average weighted attrition rates prior to commencing online treatment and after treatment have been reported to be 21%, whereas the rate was 8% from treatment completion to follow up [23].

Attrition in mental health care interventions has been shown to be up to twice as common compared to that in other medical fields [24]. For example, attrition from cognitive behavioral therapy (CBT) was reported to range from 20% to up to nearly 44%, although some indications are toward lower attrition in CBT at the group level [26]. Recent research has found that online CBT programs are effective in treating adolescent mental health problems such as anxiety and depression [5,15,25,27]. However, attrition from these programs remains an issue, with a high degree of variation and attrition rates reaching up to 50% [15,28]. To ultimately lower attrition in adolescent mental mHealth interventions, a better understanding of what factors explain mHealth usage in different adolescent subgroups and time-specific attrition is direly needed.

Adolescents with significant emotional problems (ie, anxiety and depression) were reported to have lower general self-efficacy than their peers and were more likely to either not seek treatment or drop out [3,29,30]. Originating from social cognitive theory, self-efficacy is defined as an individual’s belief that ability is sufficient to succeed or accomplish a task and has been used extensively to guide a theoretical framework for interventions targeting health behaviors [3,29-31]. Individuals with higher levels of self-efficacy are more likely to seek treatment and persist longer in their efforts to change behavior [32]. A partial reason for this appears to be effective use of self-regulatory skills such as planning, problem-solving, and self-incentives [32]. Research has shown a positive relationship between higher levels of self-efficacy and successful health behavior change in terms of weight management and exercise behavior [33]. Studies have also revealed a mediating relationship between higher levels of self-efficacy and treatment adherence in diverse chronic illnesses [34]. Attrition rates in adolescent mHealth interventions could perhaps be better accounted for by an increased understanding of the relationship between self-efficacy levels and detailed descriptions of time- and content-based usage. The purpose of this study was to assess time-specific attrition rates in an adolescent mHealth intervention, as well as to describe usage and the intervention’s feasibility in relation to self-efficacy levels and participants’ emotional and physical health.

## Methods

### Participants

Participants were 41 individuals, including 17 girls and 24 boys, between 15 and 16 years of age attending a public school in the greater capital area of Iceland. The average age at baseline was 15.6 years (SD 0.26). All children born in 2001 attending a participating public school in Iceland’s capital area were eligible participants. All participants were native Icelandic speakers and owned smartphones at baseline; 56% (n=23) of participants had smartphones operating on iOS and 44% (n=18) had those operating on Android devices. Exclusion criteria were obesity rooted in recognizable medical illness; mental retardation; physical, developmental, and mental illness significantly restricting diet or physical exercise; and not having access to an Android or iOS operating device. No participant was excluded from the study based on these criteria. Research specifications and mobile app introduction were sent to parents and legal guardians of all eligible participants through school officials by email, including a confirmative survey link along with parental information about possible exclusion criteria. Participation in the online survey was regarded as consent. The study was approved by the National Bioethics Committee (license number VSNb2015060065/03-01).

### Measurements

The primary outcome measure was app acceptability and functionality, assessed with the Systematic Usability Scale (SUS), a widely used and relatively well-studied 10-item questionnaire on app usability where the scores range from 0 to 100, and a total score over 70 indicates satisfactory usability and user acceptance [35,36]. Further primary measures were the amount, frequency, and time of daily physical activity measured through in-app activity; self-reported stress levels; and quality of sleep and energy levels, measured through levels of health app usage and completion of in-app health tasks. Cronbach α for the current sample was .73.

Self-efficacy was assessed with the General Self Efficacy Scale (GSE), a 10-item self-report questionnaire with scores ranging from 10 to 40, with a higher score yielding increases in self-efficacy [37]. GSE has shown acceptable psychometric properties in studies, and was used in global youth populations [38]. Cronbach α for the current sample was .94.

Secondary outcome measures included the standardized BMI (BMI-SDS) based on BMI index reference values for Swedish children adjusted for age and sex. Participants were weighed in kilograms in light clothing without shoes using a digital scale (Marel type C2; Marel, Reykjavik, Iceland). Height was measured in centimeters using a wall-mounted stadiometer (Seca stadiometer; Seca, Hamburg, Germany).

Participants’ depressive symptoms were assessed with Children’s Depression Inventory (CDI), a self-report assessment tool for children and youth. A T-score over 70 was used as the clinical cut-off point. The CDI’s psychometrics have been studied with acceptable findings in both US and Icelandic pediatric populations [39,40]. Cronbach α for the current sample was .82.

The Multidimensional Anxiety Scale (MASC) was used to measure anxiety symptoms. The MASC is a self-report scale with a clinical cut-off T-score over 64 and the following subscales: physical symptoms, harm avoidance, social anxiety, and separation anxiety. Acceptable psychometric properties of the MASC have been documented overall as well as in the Icelandic population [41,42]. Cronbach α for the current sample was .90.

The BEARS sleep screening algorithm was used to assess sleep problems among participants. BEARS is a screening instrument for children from 2 to 18 years old, divided into five sleep domains: bedtime problems, excessive daytime sleepiness, awakenings during the night, regularity and duration of sleep, and snoring [43]. Cronbach α for the current sample was .71.

### mHealth App

Multiple focus group studies were performed among both Icelandic public school students and adolescents in the obesity clinic at Landspitali University Hospital in Iceland to design and implement the smartphone app named SidekickHealth. Based on results from focus group studies and design advisors, the app took the form of a social health game (see Multimedia Appendix 1). Functionality is centered on helping the user set goals and create health-related missions (gamification of tasks) in three main categories: food and drink (eg, daily fruits, vegetable and water intake), physical activity (eg, body weight exercises, minutes of sports activity, GPS-based biking, walking, or running), and mental health (eg, improving sleep, reducing stress, and exercising gratitude). By completing missions and friendly competitions, the user accumulates badges, moves to higher levels, and aggregates points (called “kicks”) providing altruistic rewards (liters of water or polio vaccinations that are sent in their name to children in need through UNICEF). A visual representation of user performance in different categories is provided along with a storyline highlighting progress. Emphasis is on keeping the app fun, entertaining, and easy to use. The smartphone app operates on the Android and iOS platforms. The app’s function focuses on education and enablement through essentials of the benefits of physical activity and relaxation exercises, as well as a healthy diet, portion sizes, and appetite awareness training (AAT). AAT is a behavior tool that is used in obesity treatment, which encourages overweight/obese children and teenagers to eat in response to internal appetite cues, and has shown promise for the treatment of overweight and obese children and teenagers [44,45]; thus, AAT was visually developed as an individual mission in the app’s nutrition category. Throughout this study, mHealth usage was focused on overall health promotion in groups and individually. In weeks 2 to 4, the in-app focus was on individual mental health promotion, dietary habits, and physical exercise, respectively. Participants were randomly ascribed to health teams consisting of 6 individuals that collectively and individually competed in point collection through completion of in-app health tasks. Winners of competitions, groups and individuals, received confirmation that UNICEF had sent polio vaccinations to children in need. Further, through completion of in-app health exercises, participants collected liters of water that were sent in their name to children in need through UNICEF. The total cost for the altruistic rewards, paid for by the first author, for all in-app rewards throughout the treatment period was roughly US $16 or US $0.40 per participant.

### Procedure

**Figure 1:**
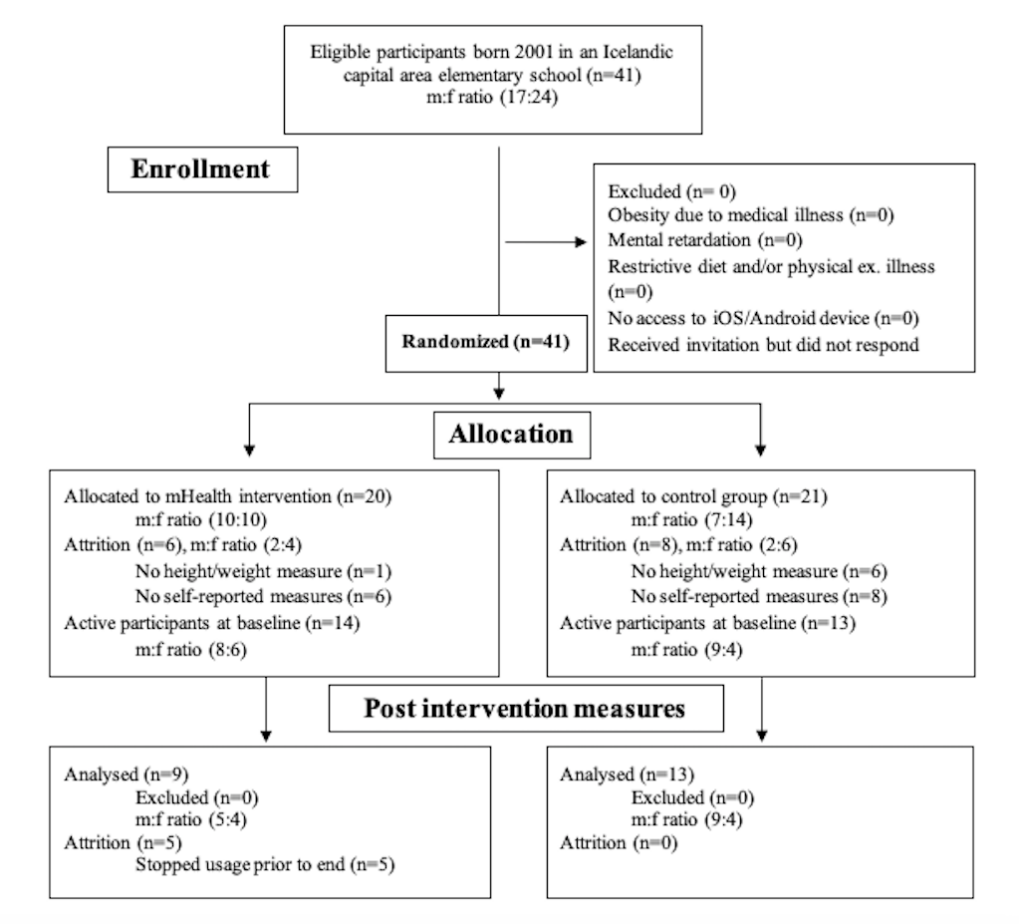
Pilot intervention flow chart. mHealth: mobile health; m: male; f: female.

The study was a randomized controlled pilot study with blind raters. The waitlist-control method with simple parallel group randomization was used to distinguish control and intervention groups. Research specifications and mobile app introduction were sent via email to the parents and legal caretakers of all eligible participants through school officials along with a confirmative survey link, which if answered yielded confirmation for participation. Measures were taken at baseline and 6 weeks later at the study end. All participants received an approximately 5-minute-long introduction regarding the study specifications. The control group received no further contact or information until study-end measures. Anthropometric measures were performed by four research assistants, all of whom were senior undergraduate students at the Psychology Department of University of Iceland. The research assistants were blinded to group assignment. The treatment group received a 10-minute introduction about the mobile app and its functions. Participants were randomly assigned with the coin toss method to teams consisting of 6 individuals that collectively and individually competed in point collection through completion of in-app health tasks. Participation in the intervention arm was defined as downloading the SidekickHealth app and completing at least 3 health exercises within the app. Weekly retention was defined as completing health exercises in the app during each week of the intervention period. Attrition rates were therefore assessed on a weekly basis. A flow chart of the study is provided in Figure 1.

### Statistical Analysis

Data are presented as means (SD) and frequency of observed behaviors. Paired-sampled *t* tests and repeated-measures analysis of variance with adjusted α levels through Bonferroni corrections were used for assessing mean treatment effects (ie, app usage, frequency of in-app exercises, and changes in BMI-SDS and health behavior variables) from baseline to post-treatment. Statistical analysis for the pilot study is mainly descriptive. Differences between population groups for categorical variables (gender, research group, mobile operating system), frequency of health behaviors (intake of fruits and vegetables, water consumption, physical activity), daily screen time, hours of sleep, and clinical cut-off rates of psychometric measures (CDI, MASC) at baseline were assessed with *χ*^2^ tests. Bidirectional correlations were assessed between predictive variables (gender, research groups, weight category); scoring in the clinical range or above the cutoff of concern on the psychological measures (CDI, MASC, BEARS, GSE); and frequency of in-app categorical health exercises and the outcome variables (treatment adherence, in-app exercises, and BMI-SDS change from baseline to post-treatment). Kendall *τ* was used to assess the relationship between app usage time categories and completion group. The variables of in-app frequency of different health category exercises (nutrition, mental health, and physical activity) were used for predicting the BMI-SDS change from pretreatment to post-treatment along with treatment adherence through standard multiple regression analyses. Data were analyzed using IBM SPSS Statistics, Release Version 26.00 (SPSS, Inc, 2009, Chicago, IL, USA).

## Results

Among all invited participants in the intervention group, 70% (male:female ratio 8:6) began the intervention. Participants’ descriptive characteristics at baseline are summarized in Table 1. Retention after 6 weeks of the intervention was 65% among those who began the intervention (male:female ratio 5:4). No significant gender difference was evident in retention rates (*χ*^2^_2, 14_=0.83, *P*=.36). The mean total score on the SUS was satisfactory (78.09, SD 9.82). There was no gender difference in total score on the SUS (*P*=.85) or in-app activity (*P*=.72), although female participants showed a higher frequency of usage on average than male participants in all health behavior categories (Table 2).

**Table 1:** Baseline characteristics of participants.

Characteristic		Control (n=21)	Intervention (n=20)
Age (years), mean (SD)		15.60 (0.26)	15.64 (0.25)
Male:female ratio		14:7	10:10
**Height and weight classification**			
	Height (m), mean (SD)	1.70 (0.74)	1.71 (0.74)
	Underweight, n (%)	4 (21)	3 (20)
	Normal weight, n (%)	14 (74)	11 (73)
	Overweight, n (%)	0 (0)	1 (7)
	Obesity, n (%)	1 (5)	0 (0)
	BMI, mean (SD)	22.01 (3.27)	21.42 (3.07)
	BMI-SDS^a^, mean	0.48	0.26
Hours of nightly sleep, mean (SD)		7.70 (1.07)	7.40 (1.71)
≥3 hours daily active screen time, n (%)		13 (92)	11 (85)
Daily consumption of vegetables, n (%)		12 (86)	9 (69)
Daily consumption of fruits or berries, n (%)		5 (36)	3 (23)
≥3 glasses daily water consumption, n (%)		9 (64)	9 (69)
Clinical anxiety symptoms, n (%)		3 (21)	5 (38)
Clinical depression symptoms, n (%)		1 (7)	2 (15)
General self-efficacy, mean (SD)		34 (4.29)	29 (6.90)

^a^BMI-SDS: standardized BMI.

**Table 2:** Weekly comparison of usage and attrition.

Week	In-app health exercises completed, n (M:F)	Attrition (%)	Active in app (%)	Individual exercises completed, mean (SD)	*P* values
1	859 (337:552)	0	100	55.25 (10.96)	N/A^a^
2	209 (60:149)	0	100	13.63 (2.94)	<.001
3	280 (142:138)	35	65	17.5 (5.33)	<.001
4	272 (193:77)	35	79	17 (6.35)	<.001
5	157 (109:48)	35	73	9.81 (4.07)	<.001
6	267 (143:124)	35	65	16.69 (8.37)	.02

^a^N/A: not applicable; all weeks were compared to week 1.

**Figure 2:**
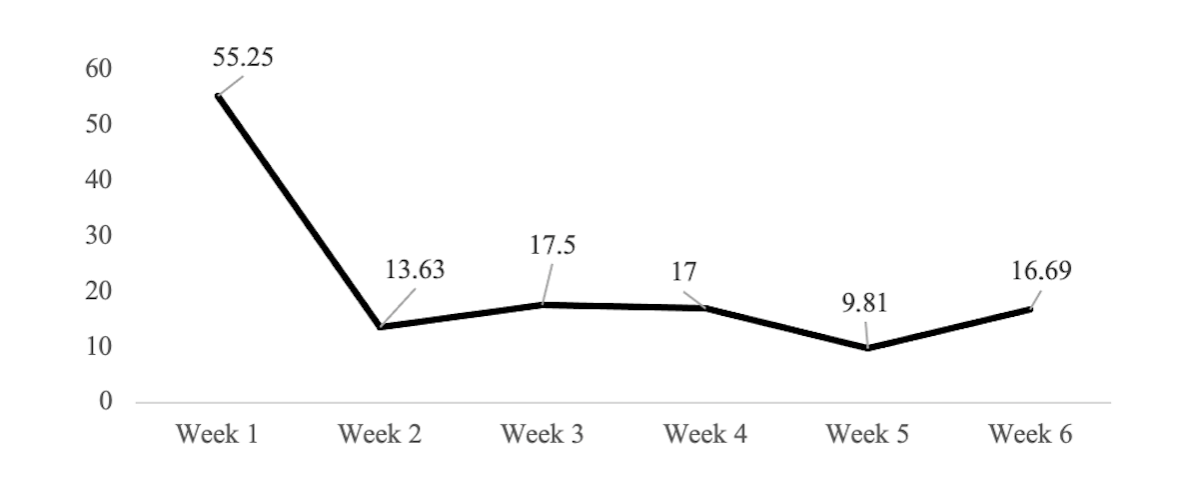
Weekly mean frequency of individual in-app exercises.

There was a significant 76% decrease in the total number of in-app health exercises from week 1 to week 2. However, from week 2 to study end, there was a 22% increase in the total number of exercises. The weekly individual mean number of in-app exercises is shown in Figure 2 and the average usage throughout the intervention is summarized in Table 2. Participants who dropped out of the intervention were significantly more likely to use the app between midnight and midday than those who completed the intervention (*R_t_*=0.43, *P<*.001). Details of when the participants used the app and the types of health exercises they completed are shown in Table 3.

**Table 3:** Times of day spent on each exercise category.

Time of usage	Food, n (%)	Physical, n (%)	Mental health, n (%)	All categories, n (%)
12 AM to 5:59 AM	42 (5.3)	40 (4.4)	24 (7.8)	106 (5.3)
6 AM to 11:59 AM	149 (18.9)	105 (11.5)	34 (11.1)	288 (14.3)
12 PM to 5:59 PM	303 (38.5)	289 (31.6)	93 (30.3)	685 (34.1)
6 PM to 11:59 PM	294 (37.3)	482 (52.6)	156 (50.8)	932 (46.3)
Total, N	788	916	307	2011

Among the 23 participants who answered the baseline questionnaire, 13 (57%) participants were either interested or very interested in using the app to increase health behavior. Four participants (17%) had never downloaded a health app prior to the intervention. However, 9 participants (39%) had never used health apps, which indicates that 5 (22%) participants had downloaded a health app that they never used. All participants had downloaded and used social media apps. Daily active screen time did not significantly differ between the intervention and control groups or between genders at baseline and at study end (*χ^2^*_1,23_=3.73, *P*=.16). At baseline, the intervention group had more problems with daytime sleepiness than the control group (*χ^2^*_5,20_=11.29, *P*=.04) but this difference was no longer detected at study end (*χ^2^*_5,23_= 2.35, *P*=.80). There were significantly greater problems with disruptive wake-ups during night sleep for participants in the intervention group than for participants in the control arm (*χ^2^*_5,20_=11.87, *P*=.04) at baseline, although no such difference was evident at study end. Mean hours of sleep did not differ significantly between the intervention group (6.90, SD 1.29) and the control group (7.15, SD 0.69; t_21_=0.4, *P*=.64) at study end.

There were no significant differences in daily portions of vegetables or fruits and berries between the intervention and control groups. There were also no differences regarding the frequency and amount of sugary or sugar-free soft drinks at pre and post measures, as well as for the consumption of salted chips, French fries, or popcorn and candy or sweets. In addition, there were no differences between the intervention and control groups in terms of the consumption of energy drinks at baseline or at study end. The perceived amount of physical exercise was increased by nearly 20% in the intervention group between baseline and study end but decreased by 26% in the control group. Total anxiety scores did not differ significantly between groups at baseline; however, an 8% decrease was found for the intervention group compared to a 4% increase in the control group (Multimedia Appendix 2). Symptoms of depression were also more evident in the SidekickHealth app intervention group at baseline. However, these differences were not apparent at study end. There was also a significant difference between the intervention group and control group in terms of negative self-esteem at baseline, which was not evident after the intervention (Multimedia Appendix 2). The total score on the GSE scale revealed a roughly 8% increase in self-efficacy scores in the intervention group compared to a 3% decrease in the control group from baseline to study end.

Correlations were found between the count of app exercises during the intervention period and weight difference from baseline to intervention end. However, none of the correlations found was statistically significant due to the small sample size. When underweight participants were excluded from the calculations, the decrease in BMI-SDS from baseline to study end appeared to be significantly more apparent in the intervention group (mean 0.48, SD 0.36) compared to that of the control group (mean 0.08, SD 0.44; t_17_=–2.14, *P*=.04).

## Discussion

There has been a steep and steady increase in mHealth interventions since their appearance roughly a decade ago, with an estimated 325,000 mHealth apps available on the market in 2017 [12]. The purpose of this study was to report attrition rates in a pilot study of an adolescent mHealth intervention and to begin to depict different attrition periods. Time-specific data alongside accurate definitions and analysis of attrition in adolescent mHealth interventions (ie, online CBT programs) targeting emotional disorders or subthreshold emotional problems in adolescent populations are lacking in the literature [15]. Studies on attrition in mHealth interventions targeting adolescent populations frequently present attrition rates in a two-point fashion, from initiation of the intervention to the end of treatment. This study offers some insight into mHealth usage among adolescents in this regard with an observed 35% attrition rate from program initiation to termination, which is similar to reported rates in previous online youth CBT programs [5]. However, as this study focused on usage and attrition rates on a weekly basis throughout the intervention, some interesting findings emerged regarding usage of the app. There was a 22% increase in usage between the second week of the intervention and termination, suggesting a sensitive attrition period shortly after instigation of mHealth interventions treating adolescents that warrants further examination. A better understanding of the timeline from treatment instigation to attrition in adolescent mHealth interventions and how emotional disorders or subthreshold emotional problems are connected to that timeline would be a reasonable next step.

Daily time of usage seemed to play a contributing role to gaining an increased understanding of attrition rates. Participants who dropped out of the intervention were significantly more likely to use the app between midnight and midday than those who completed the intervention. Those who completed the intervention were both more likely to use the app from midday to midnight as well as to complete more in-app exercises on average than those who dropped out. The latter finding may seem somewhat rudimentary but is of importance, since this indicates that participants who dropped out are not less motivated to use the app at initiation of treatment. These findings do perhaps highlight the importance of examining time of usage through survival analysis in subsequent studies assessing the factors contributing to attrition from interventions.

An integral measuring factor in the development and implementation of mHealth interventions for adolescent populations is assessing the intervention’s feasibility and usability for the desired research population. Participants reported on the app’s adequate usability on the SUS and seemed willing to engage in health exercises, completing over 21 in-app exercises on a weekly basis throughout the intervention. A significant decrease in average exercises performed between the first week of the intervention and subsequent intervention weeks was evident, although average usage leveled off at roughly 15 weekly in-app exercises throughout the intervention period. Interestingly, roughly 39% of the participants had never used an mHealth solution prior to this study, although all participants were accustomed to apps since all had downloaded and used social media apps on their smartphones. These findings reveal higher usage rates among participants as previous studies have shown that merely 8% of adolescents use health apps [13].

This study also highlights noteworthy health behavior changes based on app usage. Indications were toward a positive impact on reported sleep problems, in disruptive night sleep wake-ups, and problematic daytime sleepiness, although these findings need to be studied and documented in a more thorough manner. Reported daily physical exercise was increased by nearly 20% in the intervention group, whereas these numbers dropped by roughly 26% in the control arm. These findings could either point to the fact that in-app usage may heighten perceived levels of physical exercise or simply increase how much the adolescents are physically exercising through the intervention. This could be assessed in a more detailed manner in subsequent studies by comparing the self-reporting and frequency of actual exercise simultaneously. Both factors could aid in increasing health behavior since a perceived increase in behavior can increase self-efficacy levels among adolescents in general mHealth interventions or online CBT interventions targeting emotional problems [30]. These findings are related to the fact that self-efficacy levels increased by 8% among adolescents in the intervention arm while decreasing by 3% among controls.

In conclusion, this pilot study was designed to assess time-specific attrition rates in an adolescent mHealth intervention, as well as the usage and feasibility in relation to self-efficacy levels and participants’ emotional and physical health. The study was limited to a single research site and the results are based on a small convenience sample, which limits the ability to generalize the findings and determine the intervention’s overall efficacy. However, the results revealed interesting findings regarding sensitive attrition periods that warrant further examination. The obtained attrition rates point to a sensitive period during the first week, and indicate that adolescents who use the app in the afternoons and evenings are less likely to drop out. More research in this area seems called for as this could be studied in relation to app features such as the timing and frequency of notifications and instructions.

## Appendix

Multimedia Appendix 1 App function.

Multimedia Appendix 2 Anxiety, depression and self-efficacy measures between research groups.

## Abbreviations

AAT: appetite awareness training
BMI-SDS: standardized body mass index
CBT: cognitive behavioral therapy
CDI: Children’s Depression Inventory
GSE: General Self Efficacy Scale
MASC: Multidimensional Anxiety Scale
mHealth: mobile health
SUS: Systematic Usability Scale
