# Supplementary figures and images for: Usage and Weekly Attrition in a Smartphone-Based Health Behavior Intervention for Adolescents: Pilot Randomized Controlled Trial
Source: JMIR Form Res. 2021 Feb 17;5(2):e21432. doi: 10.2196/21432 (PMC7929738; doi:10.2196/21432)

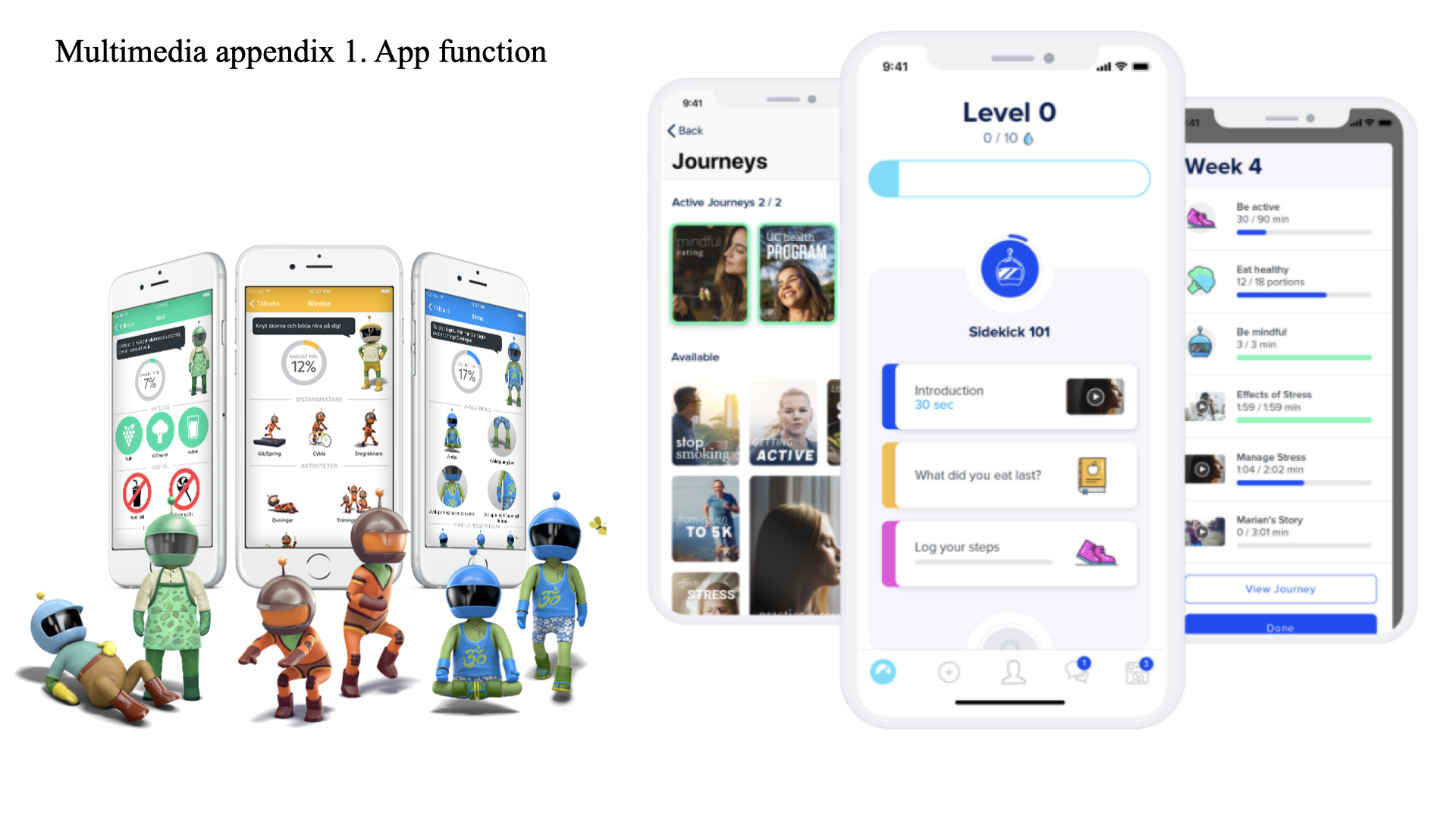

Supplement: Multimedia Appendix 1 [file formative_v5i2e21432_app1.png]
