# Supplementary material for: Usage and Weekly Attrition in a Smartphone-Based Health Behavior Intervention for Adolescents: Pilot Randomized Controlled Trial
Source: JMIR Form Res. 2021 Feb 17;5(2):e21432. doi: 10.2196/21432 (PMC7929738; doi:10.2196/21432)
Supplement: Multimedia Appendix 2 [file formative_v5i2e21432_app2.pdf]

Multimedia appendix 2

Anxiety, depression and self-efficacy measures between research groups

	Data Point	Control m(SD)	Intervention m(SD)	Sig.
Physical anxiety symptoms	Baseline	49.73 (15.93)	60.40 (10.93)	$t(19)$=-1.77
	End	56.08 (13.81)	59 (13.27)	$t(20)$=-0.51
Anxiety harm avoidance	Baseline	48.73 (10.07)	52 (11.12)	$t(19)$=-0,71
	End	48.77 (11.55)	51.30 (9.50)	$t(21)$=-0.56
Social anxiety	Baseline	44.73 (13.73)	55.90 (10.45)	$t(19)$=-2.08
	End	43.85 (10.03)	51.70 (13.74)	$t(20)$=-1.59
Separation anxiety & panic score	Baseline	54.73 (16.29)	58.50 (13.52)	$t(19)$=-0,57
	End	52.92 (14.41)	55.90 (9.72)	$t(21)$=-0,56
Total anxiety score	Baseline	48.18 (15.72)	60.60 (14.15)	$t(19)$=-1.90
	End	50.23 (13.36)	55.50 (13.10)	$t(21)$=-0,95
General self-efficacy	Baseline	34 (4,29)	29 (6,90)	$t(18)$=1,95
	End	33 (5,00)	31,60 (5,54)	$t(21)$=0,64
Depressive negative mood	Baseline	75.82 (4.14)	70.70 (6.88)	$t(19)$=2.09
	End	76.15 (7.77)	76.40 (5.21)	$t(21)$=-0.86
Depressive interpers. problems	Baseline	62.00 (6.80)	66.40 (8.30)	$t(19)$=-1.33
	End	64.23 (8.06)	66.90 (8.62)	$t(21)$=-0.76
Depressive ineffectiveness	Baseline	62.00 (6.80)	66.40 (8.30)	$t(19)$=1.27
	End	64.23 (8.06)	66.90 (8.62)	$t(21)$=1.03
Anhedonia	Baseline	55.18 (6.03)	59.90 (5.32)	$t(20)$=-1.89
	End	59.80 (6.88)	65.80 (5.94)	$t(21)$=-2.21[a]
Negative self esteem	Baseline	73.27 (5.33)	73.00 (8.11)	$t(19)$=0.92
	End	73.69 (6.47)	74.90 (10.18)	$t(21)$=-0,35
Total depression score	Baseline	69.82 (3.03)	70.40 (5.91)	$t(19)$=-0.29
	End	71.85 (7.45)	73.40 (7.03)	$t(21)$=-0.58

[a]Significant at p<0,05
